# Supplementary material for: The NCOR-HDAC3 co-repressive complex modulates the leukemogenic potential of the transcription factor ERG
Source: Nat Commun. 2023 Sep 21;14:5871. doi: 10.1038/s41467-023-41067-2 (PMC10514085; doi:10.1038/s41467-023-41067-2)
Supplement: Supplementary file 1 — Supplementary Information [file 41467_2023_41067_MOESM1_ESM.pdf]

Figure S1

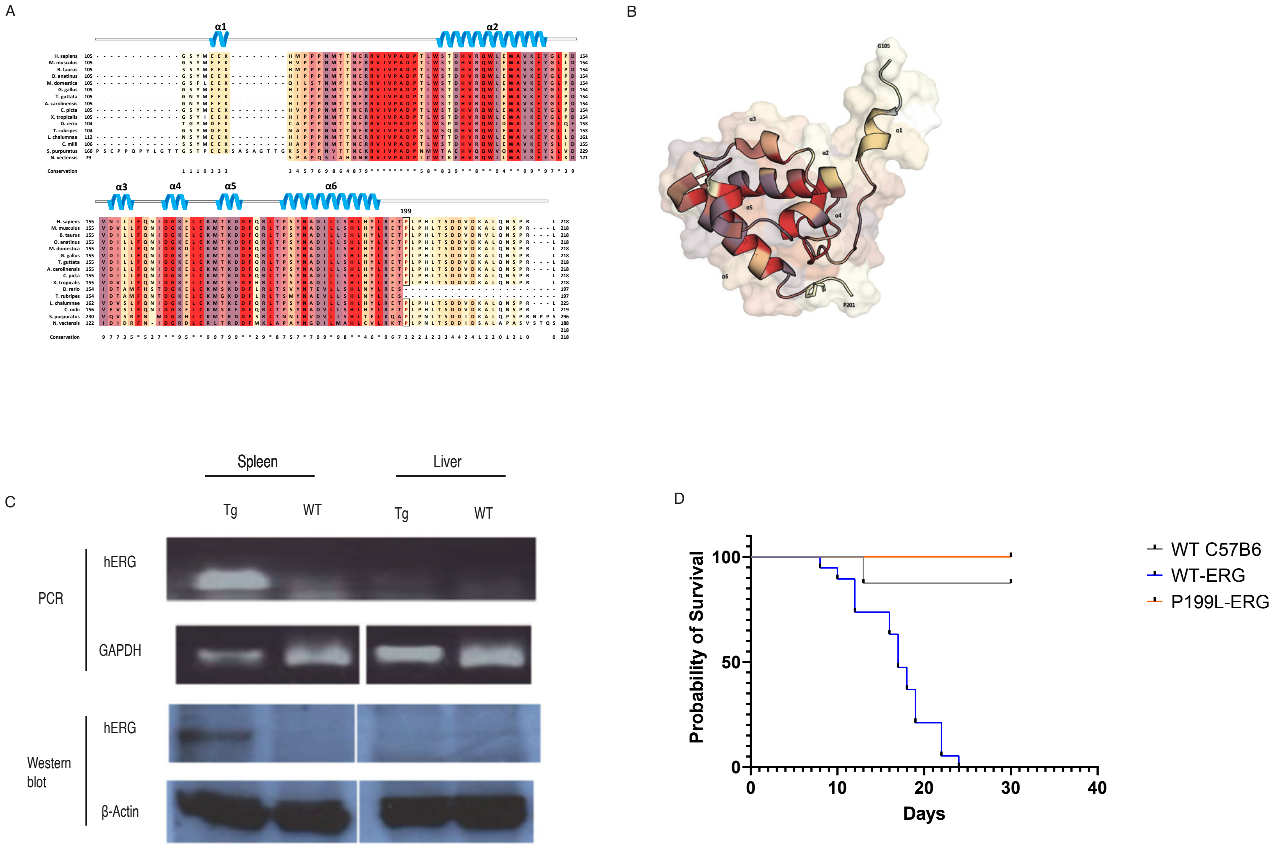

Figure S1. P199 is evolutionarily conserved in the vertebrates and inertebrates, and its P199L mutant form is not leukemogenic in transgenic mice

A. Sequence alignment of ERG from different species. Conversation scores represent the phyisc-chemical properties that are conserved in the alignment. Dark shardes of color and number indicate hgih conservation. Asterisks are colored red and indicate complete conservation. P199 is highlighted with the black boxx and red text.

B. NMR Structure (PDB: 1SXE) colored according to the degree of conservation in the alignment. Structural elements are indicated.

C. Expression of the cDNA of P199L-ERG from the spleen of the TgERG-P199L mice (upper panels) and protein (lower panels). Liver was used as a negative control.

D. Survival curve of TgERG-P199L versus TgERG-WT. Log rank test, $p < 0.0001$. TgERG- Transgenic ERG mouse.

Figure S2

A

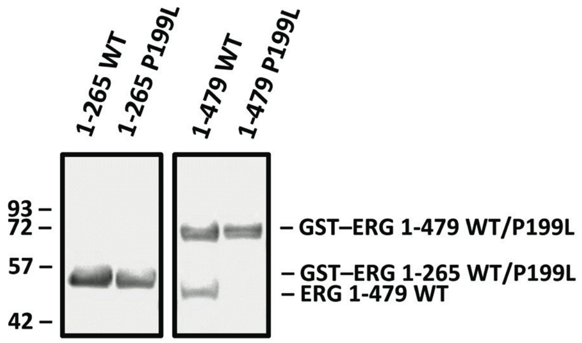

B

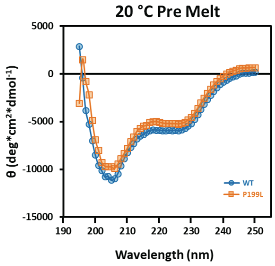

ERG 1-265	WT	P199L
α (%)	25.7	24.3
β (%)	16.9	19.3
RC	57.4	56.4

C

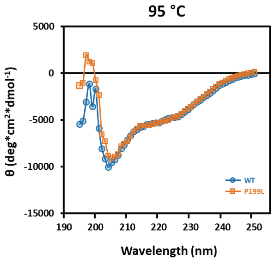

Figure S2. P199L does not significantly affect the secondary structure or thermal stability of ERG.

A. Purification of recombinant ERG proteins expressed in E.Coli BL21. Coomassive-stained SDS-PAGE of purified proteins containing N-termnial GST purification tags.
B. Circular dichroism spectrum scan at 20°C pre-melt
C. Circular dichroism spectrum scan at 95°C to denature the protein.
WT: Wildtype ERG, P199L: P199L-ERG

Figure S3

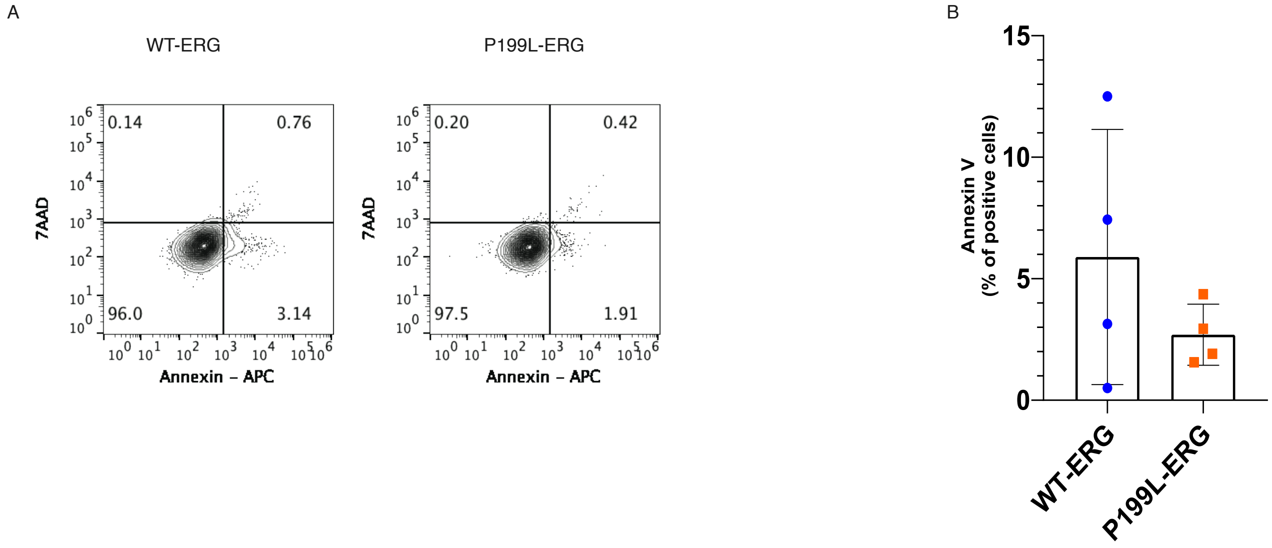

Figure S3. P199L mitigates the effect of ERG force expression on self-renewal and myeloid differentiation of HSPCs.
Apoptosis analysis of murine fetal liver derived HSPCs transduced with ERG variants using Annexin V staining. Results for round three of re-plating are shown. Representative flow cytometry analysis (left panel) and a summary for three independent experiments are shown (right panel).

Figure S4

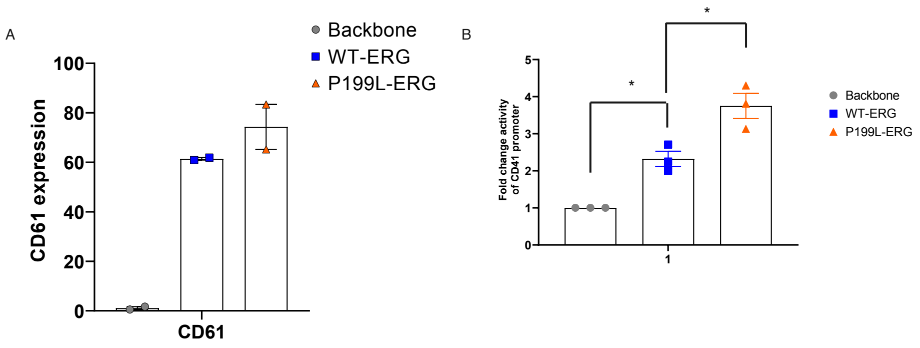

Figure S4. Induction of erythroid to megakaryocytic switch by ERG variants

A. Force expression of ERG variants in K562 cells. n=3 independent experiments in each experiment for each biological condition n=1 biological independent sample, CD61 expression was measured using flow cytometry, One-way Anova, Tukey's multiple comparison test.

B. Activity of the CD41 promoter upon expression of ERG variants in 293T cells. Fold change of luciferase expression was measured. n=3 independent experiments in each experiment for each biological condition n=1 biological independent sample, One-way Anova, Tukey's multiple comparison test, t-test, * p< 0.05, ** p< 0.01, *** p<0.001. The p-value comparing Backbone vs WT-ERG is 0.0156 and the p-value comparing Backbone vs P199L-ERG is 0.0109.

Figure S5

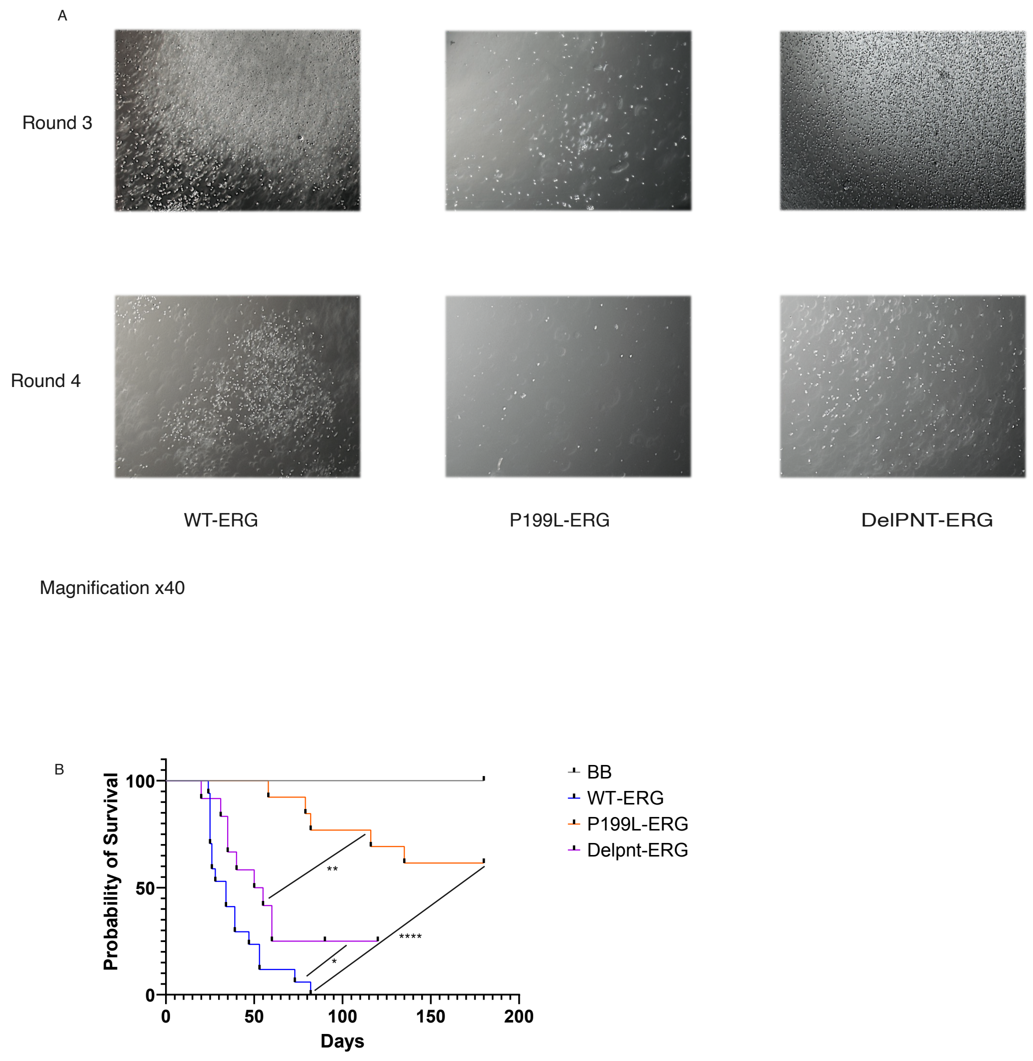

Figure S5. The phenotype of delPNT-ERG and P199L-ERG in transduction-transplantation assays in murine HSPCs.

A. Re-plating assays in semi solid conditions of fetal liver derived murine HSPCs transduced with ERG variants. CFU are presented. Media is supplemented with SCF, FLT3 and TPO.

B. Survival curve analysis of irradiated C57B/6 mice transplanted with murine fetal liver derived HSPCs following trnasduction with ERG variants (10^5 transduced cells/mouse). A long- rank test was used to compare survival distribution between groups. The p-value is p<0.0001. * p< 0.05, **p< 0.01, ***p< 0.001. The p-values comparing WT-ERG vs P199L-ERG is <0.0001, WT-ERG vs DelPNT-ERG is 0.01556 and P199L-ERG vs Delpnt -ERG is 0.00655.

Figure S6

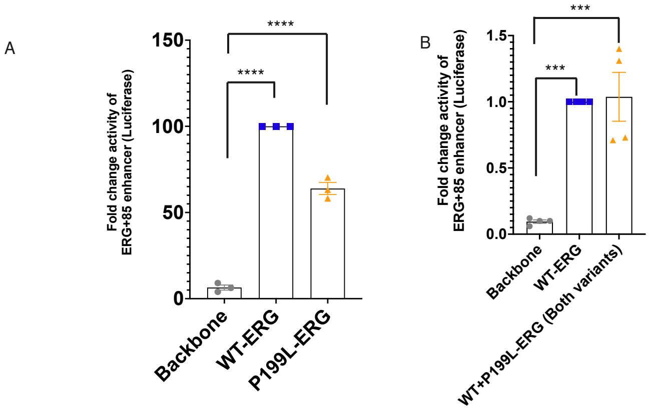

Figure S6. Activation of the ERG +85 stem cell enhancer in 293T following ERG variants force expression does not support a dominant negative role for P199L-ERG.

A. ERG+85 enhancer luciferase activity following transfection with ERG variants. n=3 independent experiments in each experiment for each biological condition n=1 biological independent sample. One-way Anova, Tukey's multiple comparsion test. The p-value comparing Backbone to WT-ERG or P199L is p< 0.0001.

B. ERG+85 enhancer luciferase activity following co-transfection of WT-ERG and WT + P199L-ERG (Both variants) in 293T cells. For the co-transfection, 500ng of each construct was used. In the case of Backbone and WT ERG alone, 1000 ng was used (grey and blue colors, respectively). n= 4 independent experiments in each experiment for each biological condition n=1 biological independent sample. One-way Anova, Tukey's multiple comparsion test. The p-value comparing Backbone to WT-ERG or P199L is p< 0.0005. denotes p< 0.05, * denotes p< 0.01, *** denotes p<0.001, **** denotes p <0.0001

Figure S7

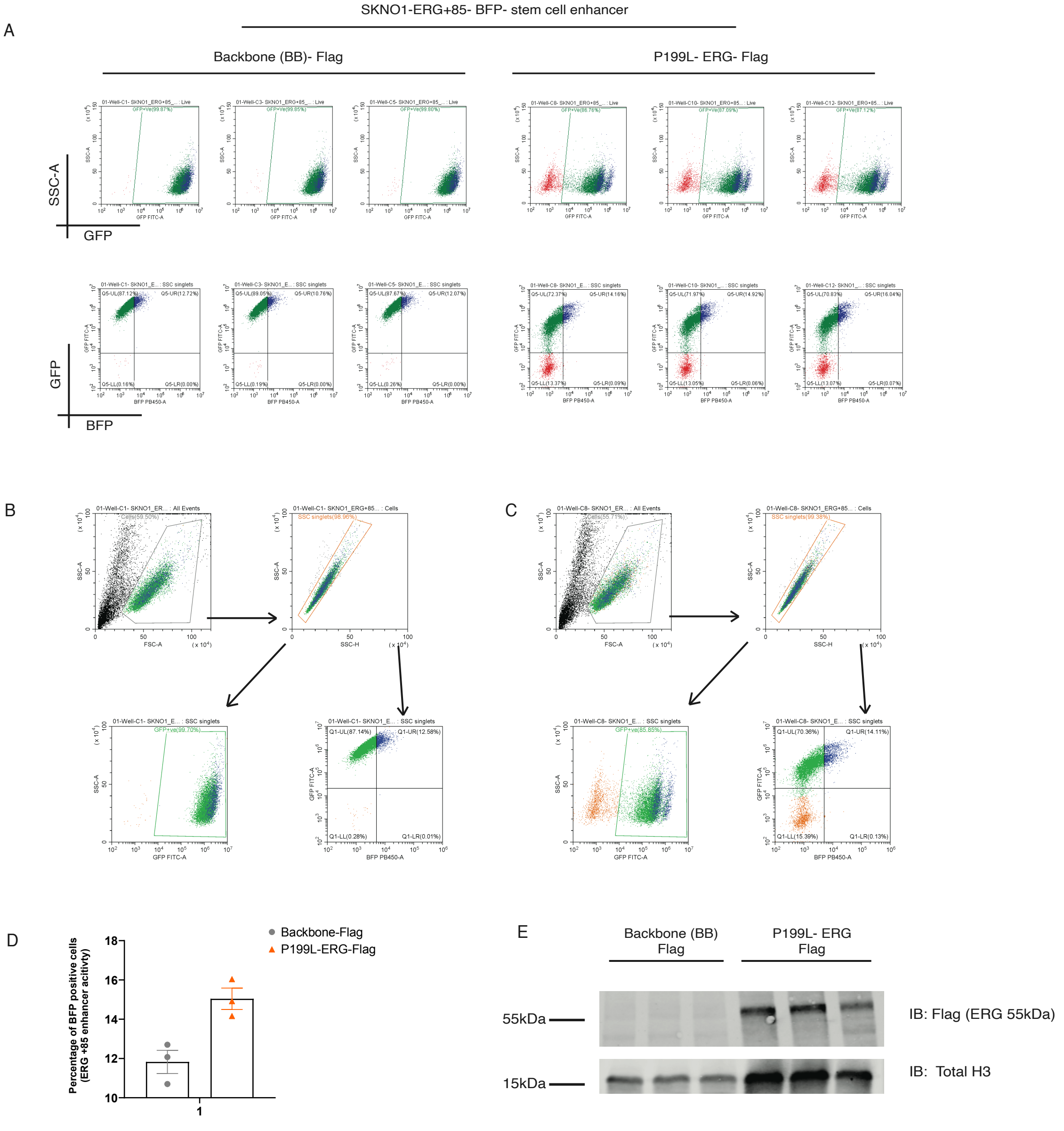

Figure S7. Activation of ERG+85 stem cell enhancer reporter in ERG dependent SKNO1 cells following forced expression of P199L-ERG.

A. Upper line- GFP marks transduced cells stably expressing the reporter. Bottom line- BFP marks the activation of the ERG+85 enhancer.
B. Gating strategy for SKNO1-ERG+85-BFP cells transduced with backbone-flag.
C. Gating strategy for SKNO1-ERG+85-BFP cells transduced with P199L-flag.
D. Summary of the three independent experiments. p= 0.016, t-test.
E. Western blot in SKNO1 cells transduced with P199L-ERG and backbone for control. n=3 independent experiments in each experiment for each biological condition n=1 biological independent sample.

Figure S8

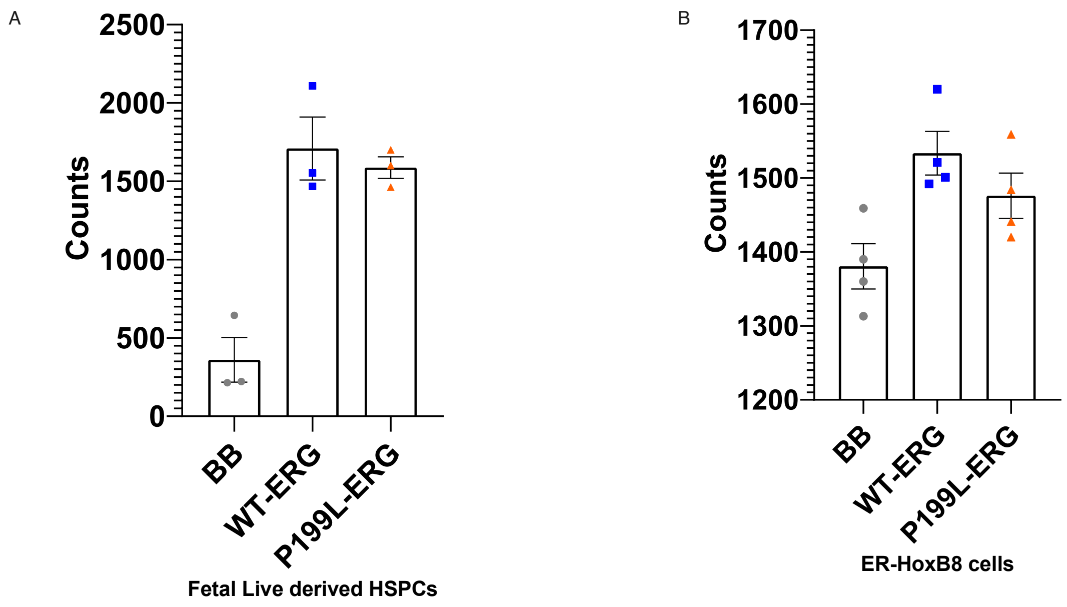

Figure S8: Expression of endogenous Erg is similar in murine HSPC following WT and P199L-ERG force expression.

A. Expression counts obtained from RNA sequencing of fetal liver derived HSPC cells expressing ERG variants.
B. Expression counts obtained from RNA sequencing of ER-HoxB8 cells expressing ERG variants.
Normalized counts are presented.

Figure S9

A

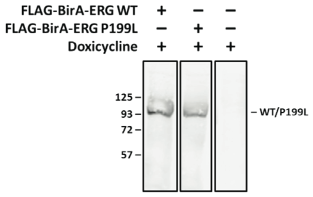

FLAG – BirA* ERG 1–479	Technical Replicate 1	Technical Replicate 2
WT	8433	8740
P199L	7088	7298

B

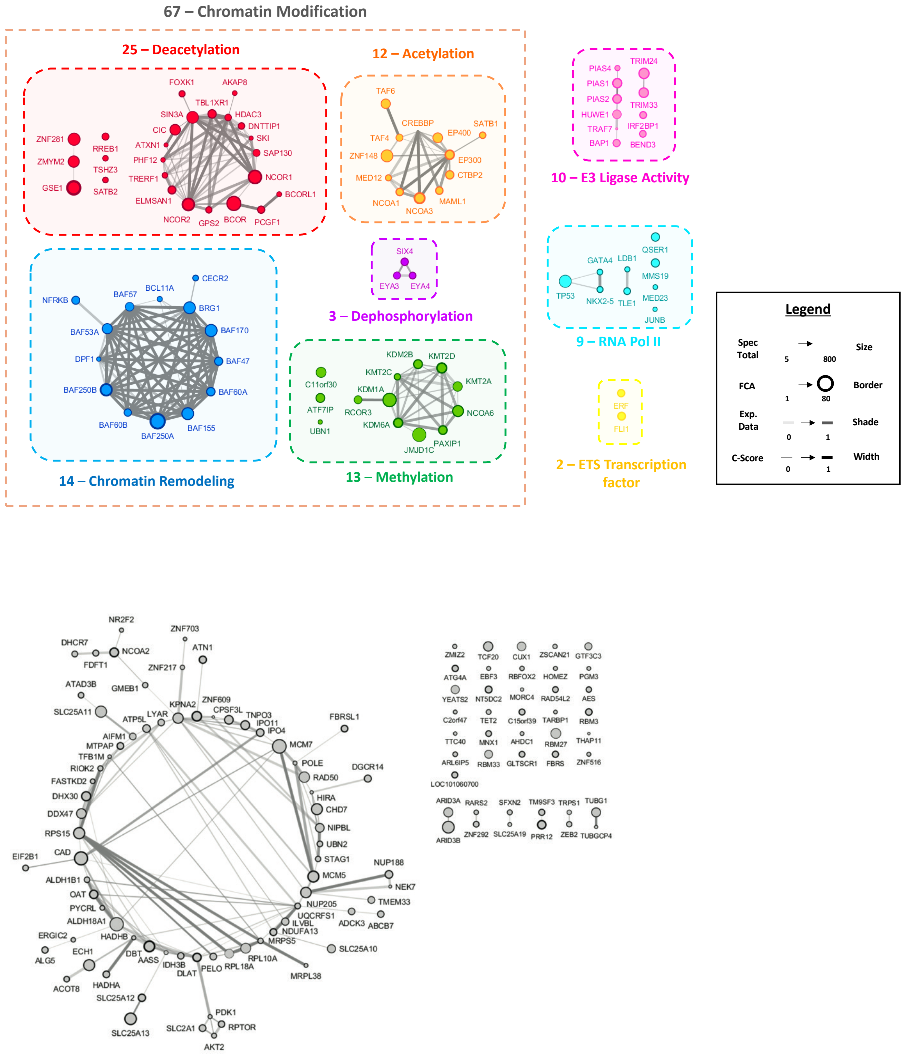

Figure S9. P199L disrupts the interaction of ERG with chromatin modifiers complexes.

A. Anti-BirA western blot of HEK293 Gnt -/- cell pellets for BioID.

B. The complete network of identified proximal ERG interactors. Proteins are grouped and colored according to GO molecular function. The size of the nodes is weighted by total spectral count, the width of the node boarder is weighted by the FCA score, the shade of the connecting lines is weighted by the experimental/ biochemical evidence of the interaction data and the width of the connecting line is weighted by a combined score (C- Score) that factors in phylogenetic co-ocurrence, co-expression and experimentally determined interaction between the two connected nodes.

Table S1 The proximitome of WT and P199L-ERG.

WT Only	WT AVG (SC/run)	WT (Unique peptides)/run	WT (% coverage)	P199L AVG (SC/run)	% change (P199L/WT)	P199L SPe	P199L bFDR	Function
BCL11A	8.5	4	11.95	2	-76.47	0.27	0.27	BAF superfamily
CECR2	11	2.5	2.9	6.5	-40.91	0.5	0.11	BAF superfamily
BEND3	15.5	7	12	4	-74.19	0.55	0.07	E3 Ligase
ERF	19.5	5	21.55	2.5	-87.18	0.5	0.13	ETS
EP300	42.5	10	8.55	15.5	-63.53	0.5	0.09	Histone acetyltransferase
TAF6	38	9.5	19	14.5	-61.84	0.28	0.27	Histone acetyltransferase
MAML1	21.5	8	18.3	9	-58.14	0.2	0.29	Histone acetyltransferase
HDAC3	14	3.5	15.2	3.5	-75.00	0.15	0.32	Histone deacetylase
DNTTIP1	14	4	21.6	4.5	-67.86	0.5	0.15	Histone deacetylase
GPS2	13	2.5	21.1	6.5	-50.00	0.5	0.11	Histone deacetylase
TRERF1	10	6	10.2	2.5	-75.00	0.5	0.13	Histone deacetylase
FOXK1	12.5	5	10.6	6	-52.00	0.5	0.1	Histone deacetylase
RREB1	16.5	4.5	6.9	6	-63.64	0.67	0.04	Histone deacetylase
SIN3A	88	21	25.9	60	-31.82	0	0.58	Histone deacetylase
BCORL1	7.5	3	2.4	4.5	-40.00	0.48	0.2	Histone deacetylase
MED12	11.5	5.5	4.05	4	-65.22	0.2	0.29	Histone methyltransferase
KMT2C	13.5	4	3	5	-62.96	0.58	0.05	Histone methyltransferase
JMJD1C	166	34.5	23.4	89	-46.39	0	0.55	Histone methyltransferase
RCOR3	21	7	32.2	11	-47.62	0.4	0.23	Histone methyltransferase
LDB1	8.5	3	20.15	3	-64.71	0.5	0.15	RNA polymerase II complex
QSER1	26.5	12	13.65	11	-58.49	0.01	0.43	RNA polymerase II complex
ATG4A	20	2	11.6	2.5	-87.50	0.49	0.18	Other
RAD54L2	16	6	6.35	2.5	-84.38	0.25	0.28	Other
ZNF292	15	7.5	4.65	2	-86.67	0.35	0.25	Other
SLC2A1	15	3.5	8	6.5	-56.67	0.23	0.29	Other
ARID3A	60	11.5	36.6	20	-66.67	0	0.45	Other
YEATS2	39	14.5	16.55	13.5	-65.38	0	0.53	Other
TET2	10	4.5	4.45	2.5	-75.00	0.5	0.14	Other
ZSCAN21	8	2	8.45	3	-62.50	0.29	0.26	Other
UBN2	10	4.5	4.75	5	-50.00	0.5	0.11	Other
ZEB2	12	4	4	6.5	-45.83	0.13	0.32	Other
FDFT1	11.5	3	10.3	6	-47.83	0.4	0.23	Other

WT and P199L	WT AVG (SC/run)	WT (Unique peptides)/run	WT (% coverage)	P199L AVG (SC/run)	% change (P199L/WT)	P199L SPe	P199L bFDR	Function
BAF250B	120.5	30.5	25.95	68	-43.57	1	0	BAF superfamily
BAF155	108.5	16.5	23.3	71.5	-34.10	1	0	BAF superfamily
BAF250A	392	48.5	38.65	238.5	-39.16	1	0	BAF superfamily
BAF60B	19.5	7	25.8	9.5	-51.28	1	0	BAF superfamily
TRIM33	45.5	12	14.1	28.5	-37.36	1	0	E3 Ligase
NCOA3	76	21	23.9	23.5	-69.08	1	0	Histone acetyltransferase
ZNF148	97.5	15	35.15	53.5	-45.13	0.81	0.02	Histone acetyltransferase
NCOR1	189	36.5	28.95	114	-39.68	1	0	Histone deacetylase
ZMYM2	76.5	20	25.7	52.5	-31.37	1	0	Histone deacetylase
CIC	61.5	13	18.25	37.5	-39.02	1	0	Histone deacetylase
BCOR	238	38.5	37.35	144.5	-39.29	1	0	Histone deacetylase
ZNF281	103.5	20	46.65	77.5	-25.12	1	0	Histone deacetylase
GSE1	259	22.5	40.95	173	-33.20	1	0	Histone deacetylase
KMT2D	134.5	29.5	21.65	61.5	-54.28	1	0	Histone methyltransferase
ATF7IP	33.5	9	12.35	21.5	-35.82	1	0	Histone methyltransferase
KDM1A	184.5	20.5	40.65	127.5	-30.89	1	0	Histone methyltransferase
KMT2D	56.5	24.5	10.05	27.5	-51.33	1	0	Histone methyltransferase
TCF20	67.5	20.5	22.9	55	-18.52	1	0	Other
PRR12	54	14	15	22	-59.26	1	0	Other
THAP11	3.5	2	5.4	6.5	+85.71	1	0	Other
CHD7	70.5	21	11.95	35	-50.35	1	0	Other

Table S1 (continued) The proximitome of WT and P199L-ERG.

P199L Only	P199L AVG (SC/run)	P199L (Unique peptides)/run	P199L (% coverage)	WT AVG (SC/run)	% change (P199L/WT)	WT SPe	WT bFDR	Function
ESRRA	11.5	3	8.4	0	–	–	–	Other
BCKDK	5	2.5	11.5	1	+400.00	0.46	0.21	Other
ATP1B3	11	3.5	18.45	4.5	+144.44	0.5	0.14	Other
MRPL39	10	2.5	10.65	4.5	+122.22	0.5	0.1	Other
ADNP2	4.5	2.5	2.3	1.5	+200.00	0.49	0.18	Other
MDN1	12	6	1.55	6	+100.00	0.67	0.04	Other
UTP6	5	3	8.55	2	+150.00	0.5	0.15	Other
PNO1	9.5	2.5	14.3	5	+90.00	0.5	0.17	Other
YARS2	11	5.5	20.9	6	+83.33	0.13	0.32	Other
MECOM	11.5	4	6.4	6.5	+76.92	0.5	0.09	Other
KIAA1279	14.5	4.5	15.3	8.5	+70.59	0.29	0.26	Other
LRRC40	57.5	15.5	41.2	42.5	+35.29	0.12	0.33	Other
DNAJA3	47	7.5	19.75	36	+30.56	0.15	0.32	Other
MRPL3	4	2	6.5	2.5	+60.00	0.5	0.13	Other
TMED10	15	4	18.3	11.5	+30.43	0.39	0.24	Other
IARS2	18	7	7.6	14	+28.57	0.72	0.03	Other
PHF14	5	2	4.8	3.5	+42.86	0.5	0.11	Other
STT3B	11	5	7.4	8.5	+29.41	0.78	0.02	Other
RPL7L1	14	4.5	21.95	11.5	+21.74	0.64	0.04	Other
DLD	48.5	5	19.75	41.5	+16.87	0.77	0.02	Other

Figure S10

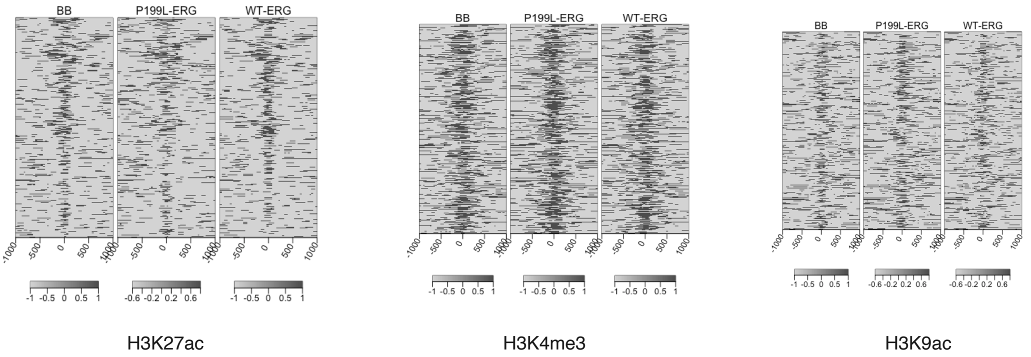

Figure S10. H3K27ac signature does not significantly differ between constructs at regions associated with transcription start sites.

ChIP sequencing analysis of ER-HoxB8 cells. Heatmap hierarchical clustering centered on H3K27ac peaks associated with differentially expressed and repressed genes following WT-ERG force expression in ER-HoxB8 cells. Co-occupancy regions of H3K27ac, H3K4me3 and H3K9ac marks is shown. BB- Backbone.

Figure S11

A

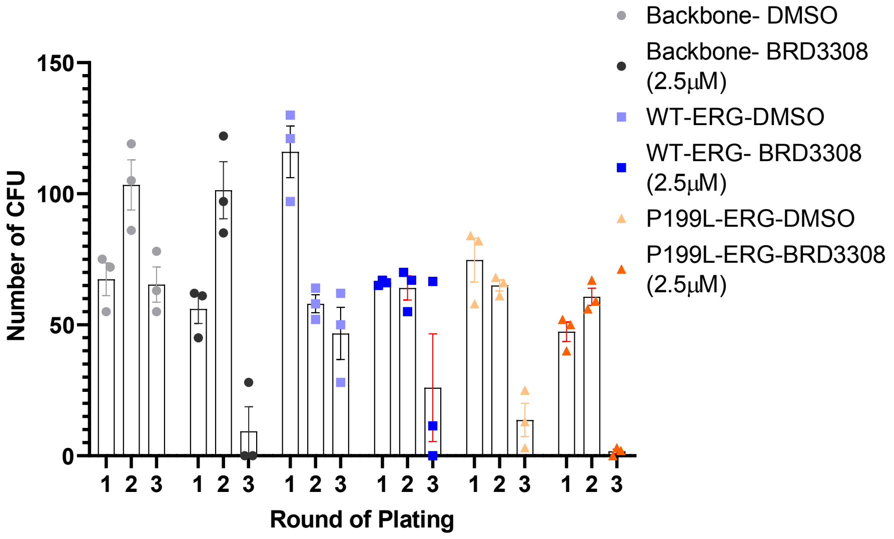

B

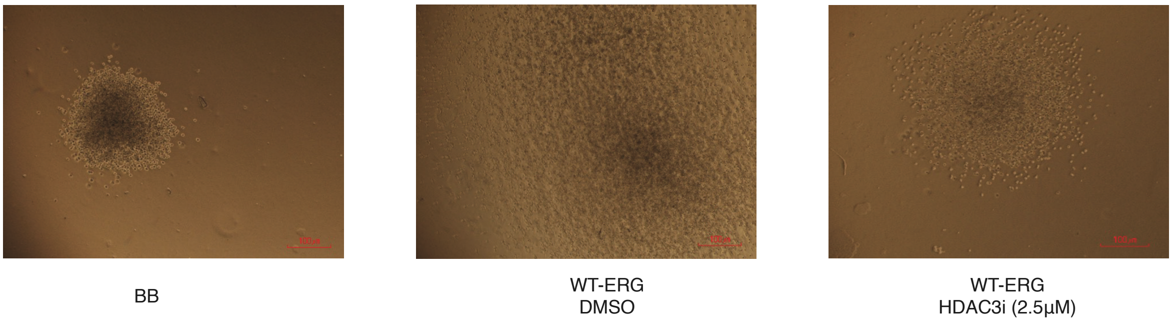

Figure S11. HDAC3 inhibition disrupts the self-renewal capacity of HSPCs over-expressing ERG.
Re-plating assay in semi-solid condition of murine fetal liver derived HSPCs (E13.5) transduced with ERG variants (media was suipplemented with IL3, IL6, SCF, 10^4 cells in each round). Cells were treated on the day of plating with either 2.5μM BRD3308 or DMSO for control. A summary of four independent experiments is shown.

A. Number of CFU per 10^ 4 cells plated is presented for each round of plating (Data are represented as means ± SEM,).
B. Microscopic view of colonies morphology between groups. Magnification 40x.

Tabke S2.

Differentiation		ENSMUSG00000015053.11	ENSMUSG00000040732.15	ENSMUSG00000030786.15	ENSMUSG00000057666.15
Hours	Replicate	GATA2	ERG	ITGAM (CD11b)	GAPDH
0	A	3023	3632	176	65672
0	B	3368	4106	236	75793
4	A	5199	4012	159	111028
4	B	4283	3681	172	93035
8	A	7487	3615	286	86209
8	B	6703	3465	250	77798
12	A	5916	3487	700	72325
12	B	5358	3299	596	80049
24	A	3857	3018	1353	74852
24	B	4264	3219	1534	92318
36	A	2798	3195	5671	75378
36	B	3235	2935	5143	93095
48	A	2362	3191	19213	74978
48	B	2498	3361	15612	70729
72	A	1296	2572	62135	46954
72	B	1382	2469	48072	52041
96	A	514	1999	106734	40107
96	B	569	2445	104976	41652
120	A	310	651	97810	45616
120	B	325	459	63033	36412

Table S2: Normalized read counts for mouse Gata2, Erg, Gapdh and Cd11b uring the 5-day differentiation time course of ER-HoxB8 cells. RNA-seq A and B stand for two different replicates. Courtesy of Dr. David Sykes.

Figure S12

A

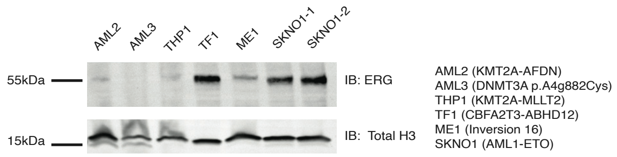

B

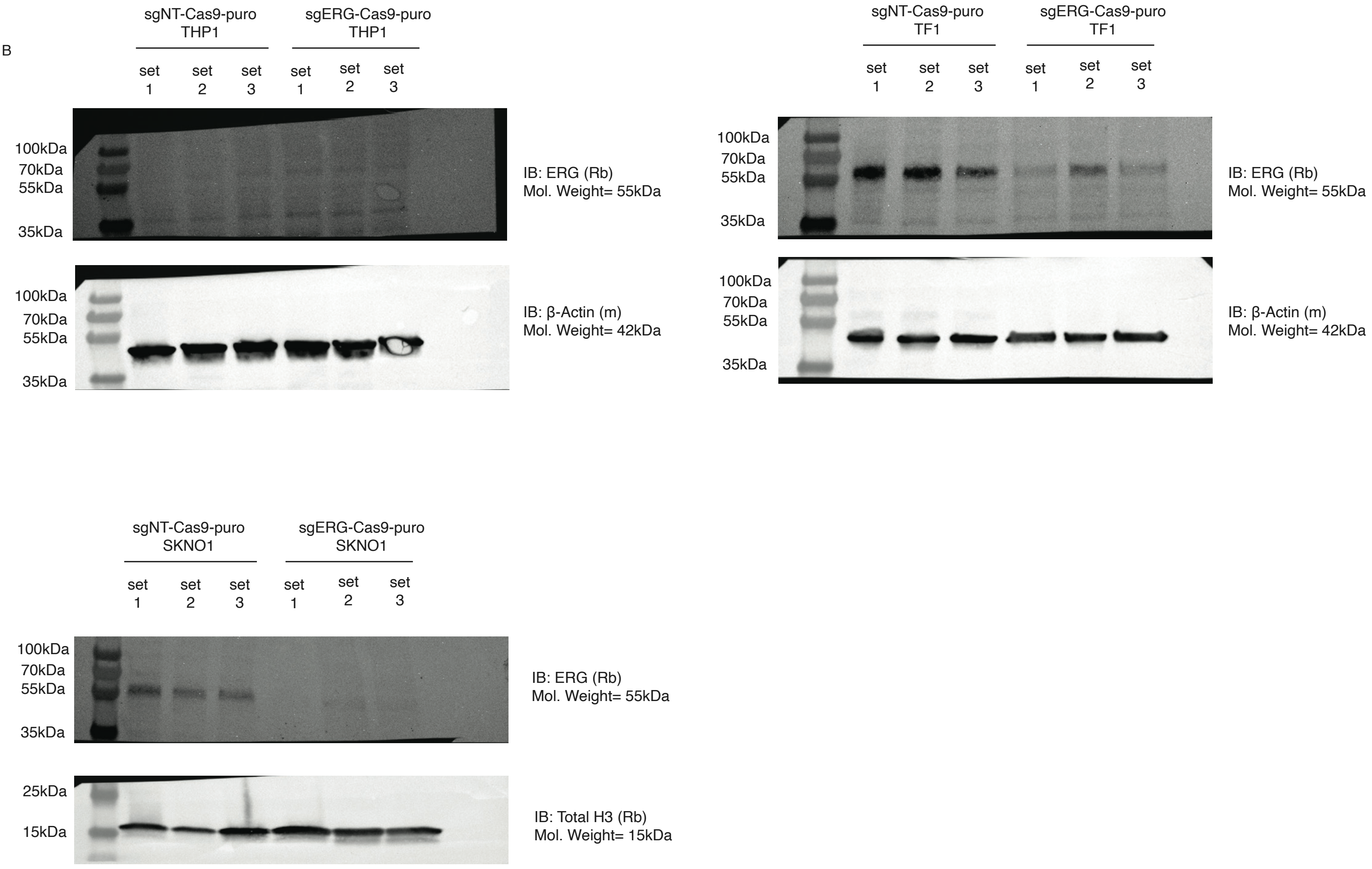

C

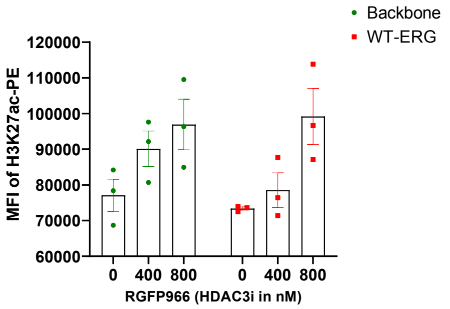

Figure S12. HDAC3 inhibition attenutaed ERG-dependent human AML proliferation.

A. Immunoblot for human ERG in human AML lines.
B. Verification of ERG knockout in THP1, TF1 and SKNO1 ells using CRISPR-Cas9 system (upper panel- immunoblot for ERG, lower panel- immunoblot for human β-Actin).
C. Western blot in SKNO1 cells transduced with P199L-ERG and backbone for control.

Figure S13

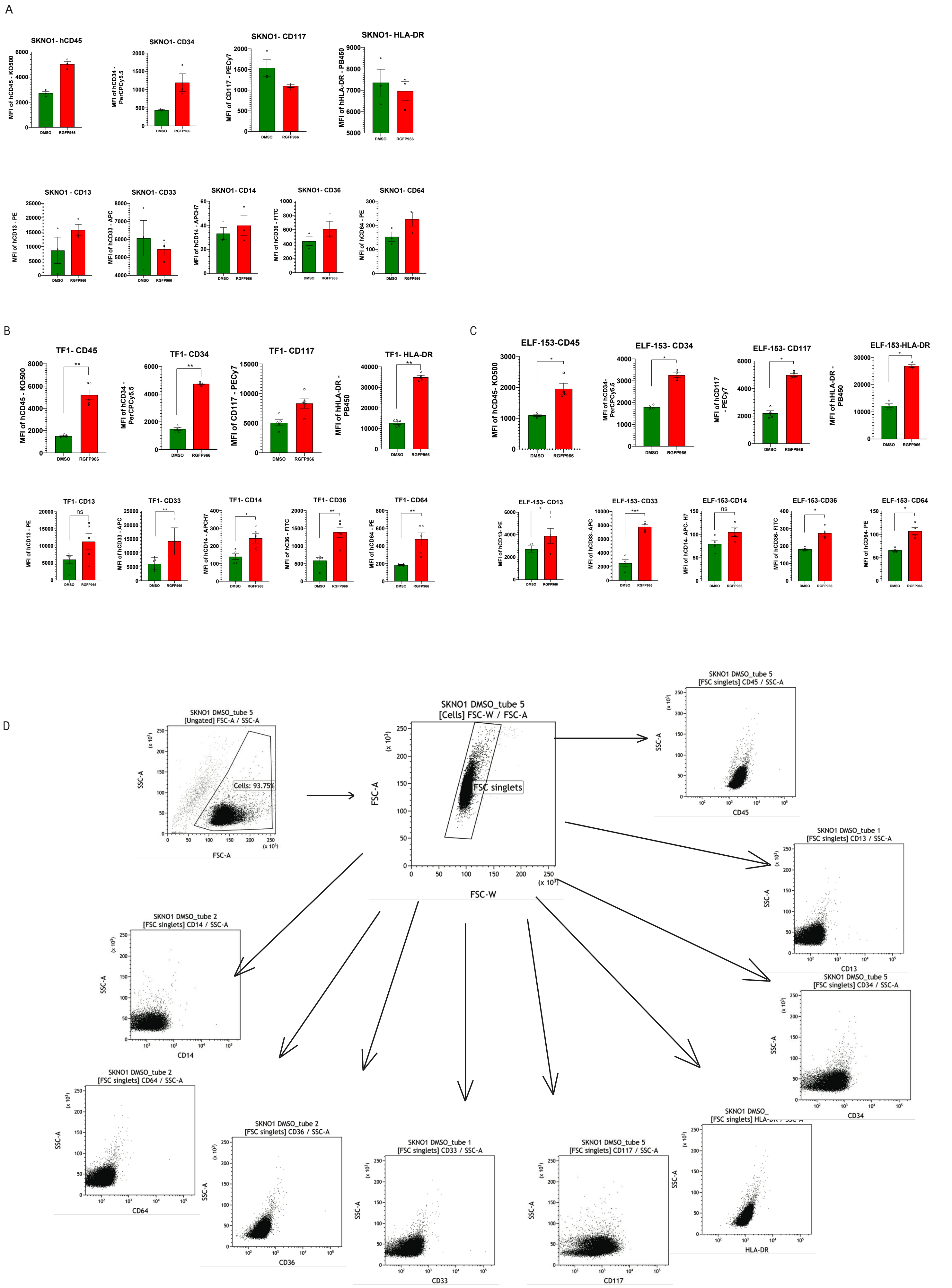
A
SKNO1- hCD45
SKNO1- CD34
SKNO1- CD117
SKNO1- HLA-DR
SKNO1 - CD13
SKNO1- CD33
SKNO1- CD14
SKNO1- CD36
SKNO1- CD64
DMSO
RGFP966
B
TF1- CD45
TF1- CD34
TF1- CD117
TF1- HLA-DR
TF1- CD13
TF1- CD33
TF1- CD14
TF1- CD36
TF1- CD64
C
ELF-153-CD45
ELF-153- CD34
ELF-153- CD117
ELF-153-HLA-DR
ELF-153- CD13
ELF-153- CD33
ELF-153-CD14
ELF-153-CD36
ELF-153- CD64
D
SKNO1 DMSO_tube 5
[Ungated] FSC-A / SSC-A
Cells: 93.75%
[Cells] FSC-W / FSC-A
FSC singlets
FSC-W
[FSC singlets] CD45 / SSC-A
[FSC singlets] CD13 / SSC-A
[FSC singlets] CD34 / SSC-A
[FSC singlets] CD14 / SSC-A
[FSC singlets] CD64 / SSC-A
[FSC singlets] CD36 / SSC-A
[FSC singlets] CD33 / SSC-A
[FSC singlets] CD117 / SSC-A

Figure S13

E

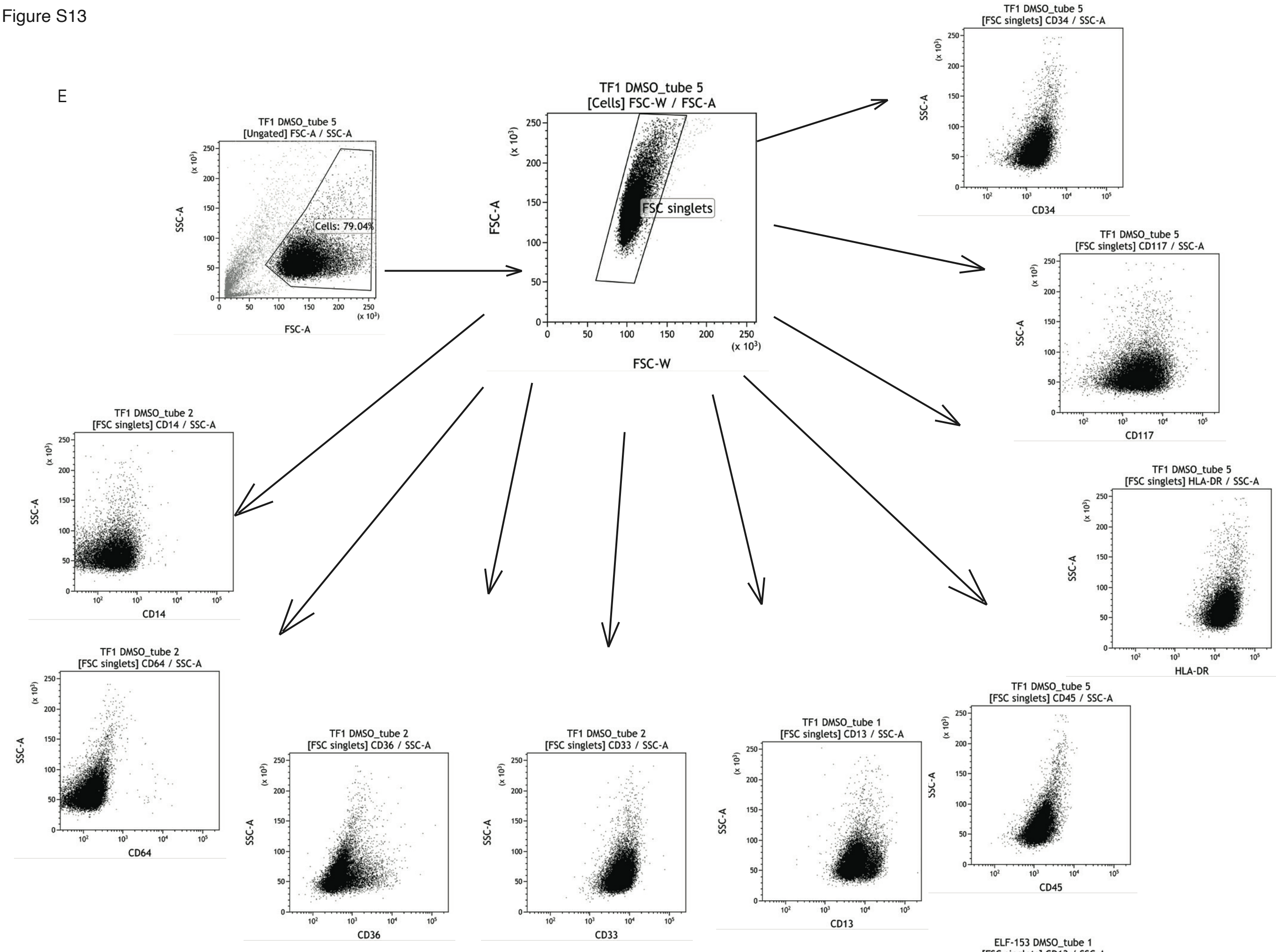

F

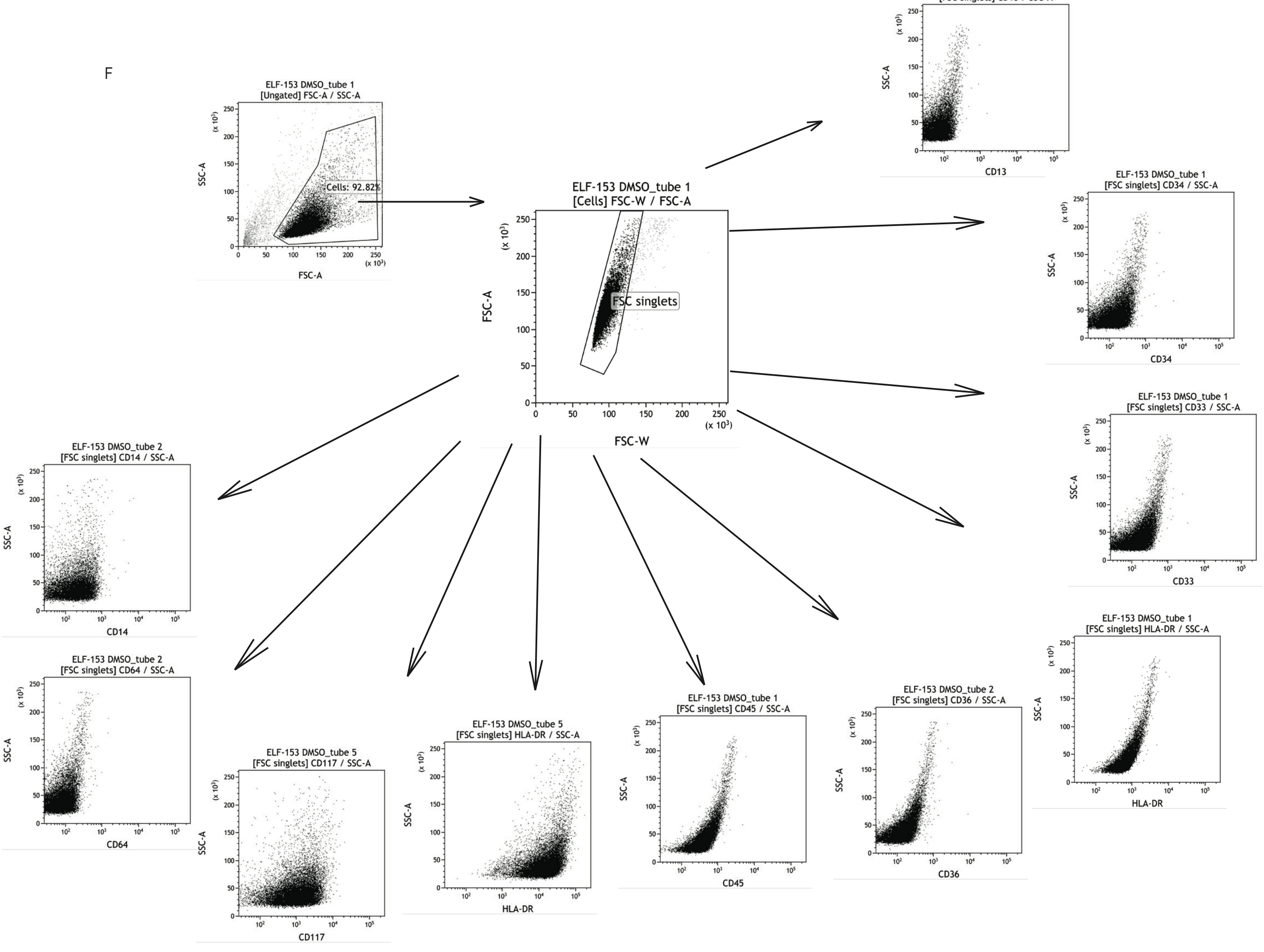

Figure S13

Figure S13. Flow cytometry analysis of ERG dependent human AML cell lines following pharmacological HDAC3 inhibition.

A. Immunophenotype was assessed for SKNO1 cells following treatment with RGFP966 for 96 hours. n= 3 independent biological replicates.
B. Immunophenotype was assessed for TF1 cells following treatment with RGFP966 for 96 hours. n= 3 independent biological replicates.
C. Immunophenotype was assessed for ELF-153 cells following treatment with RGFP966 for 96 hours. n= 3 independent biological replicates
D. Gating strategy used to analyse the mean fluorescent intensity of each fluorephore in SKNO1 cells treated with either DMSO or RGFP966.
E. Gating strategy used to analyse the mean fluorescent intensity of each fluorephore in TF1 cells treated with either DMSO or RGFP966.
F. Gating strategy used to analyse the mean fluorescent intensity of each fluorephore in ELF-153 cells treated with either DMSO or RGFP966.

Figure S14

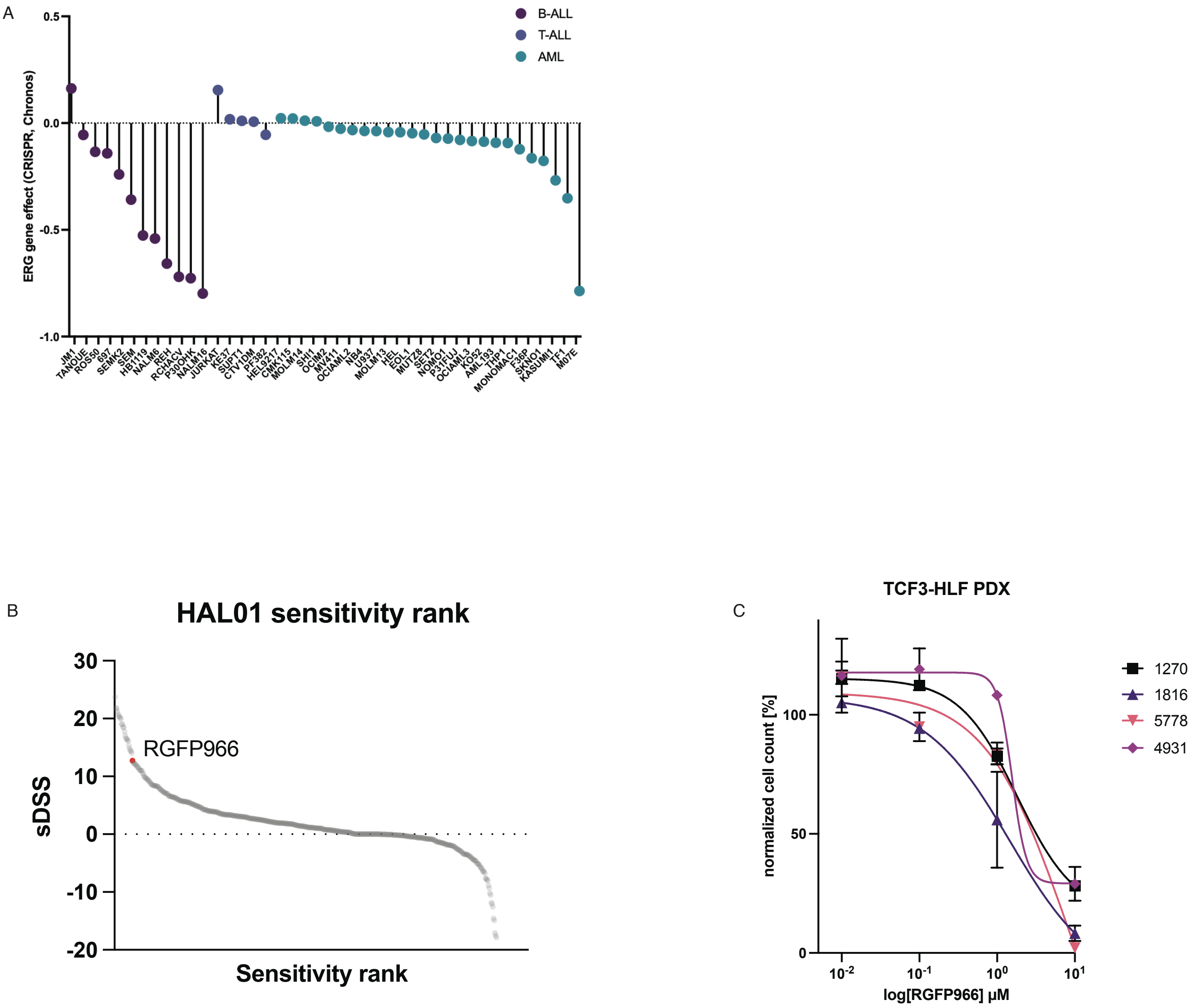

Figure S14. TCF3-HLF ALL sensitivity to HDAC3 inhibition.

A. DepMap data demonstrating ERG dependency in various leukemia cell lines.
B. Selective drug sensitivity score (sDSS) for HAL01 cell line retrieved from the FORALL portal (https://proteomics.se/forall/.).
C. TCF3_HLA PDX treated for 6 days with RGFP966 (HDAC3 inhibitor) (ex-vivo.)

Figure S15

A

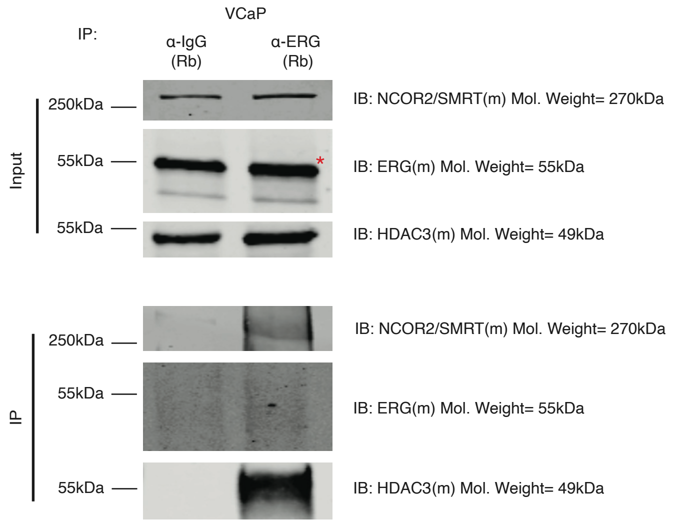

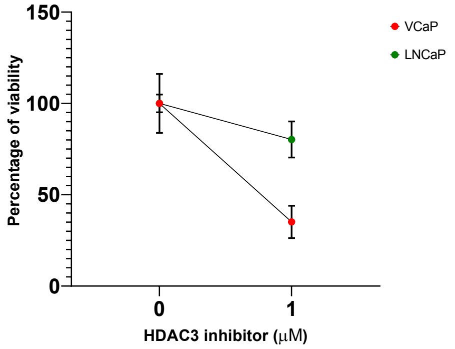

A

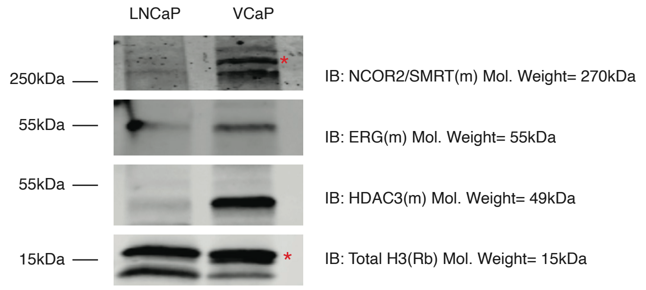

Figure S15: ERG driven prostate cancer cells are sensitive to HDAC3 inhibition.

A. Co-IP for endogenous ERG and the NCoR-HDAC3 complex.
B. HDAC3 inhibition using RGFP966 (HDAC3 inhibitor) in VCaP and LNCaP cells. Live cells were counted using flow cytometery. n= 3 independent biological experiments
C. Immunoblot for ERG and NCoR-HDAC3 proteins in VCaP and LNCaP cells. Red asterisk marks correct position of the band according to kDa estimation.

Figure 16

A

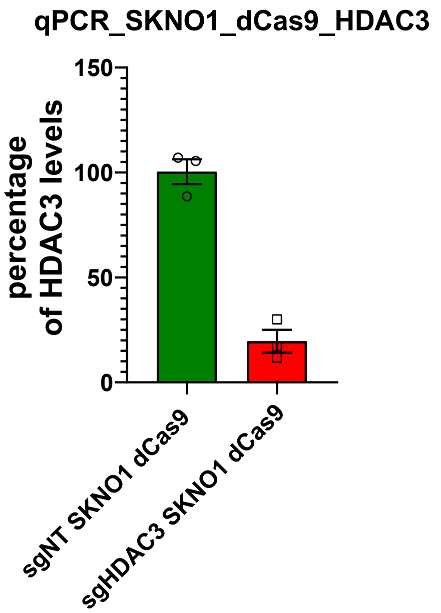

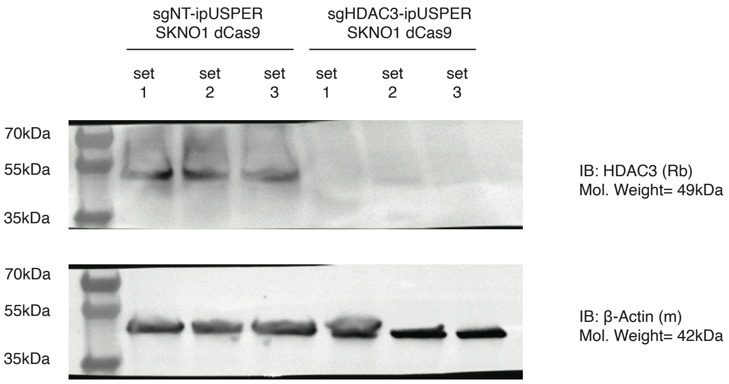

B

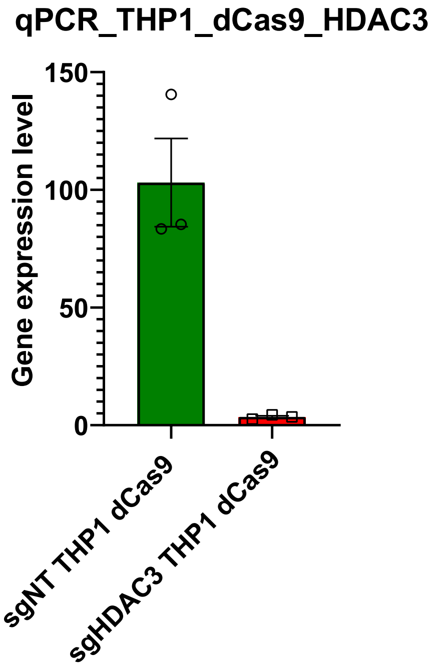

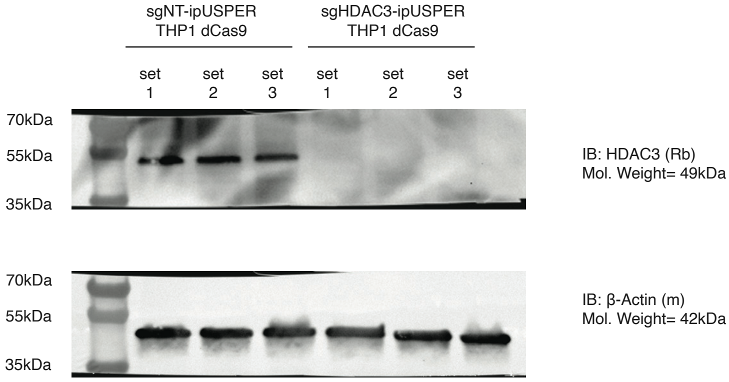

Figure S16: CRISPR-dCas9 targeting of HDAC3 expression in SKNO1 and THP1 cells.

A. qPCR (left panel) and immunoblot for human HDAC3 (right panel) in SKNO1 cells. Approximately 75-80% decrease in HDAC3 signal was measured in three sets.
B. qPCR (left panel) and immunoblot for humaan HDAC3 (right panel) in THP1 cells.
n=3 independent biological experiments
