# Supplementary material for: The NCOR-HDAC3 co-repressive complex modulates the leukemogenic potential of the transcription factor ERG
Source: Nat Commun. 2023 Sep 21;14:5871. doi: 10.1038/s41467-023-41067-2 (PMC10514085; doi:10.1038/s41467-023-41067-2)
Supplement: Supplementary file 3 — Reporting Summary [file 41467_2023_41067_MOESM3_ESM.pdf]

nature portfolio

Corresponding author(s): Shai Izraeli

Last updated by author(s): Aug 3, 2023

Reporting Summary

Nature Portfolio wishes to improve the reproducibility of the work that we publish. This form provides structure for consistency and transparency in reporting. For further information on Nature Portfolio policies, see our Editorial Policies and the Editorial Policy Checklist.

Statistics

For all statistical analyses, confirm that the following items are present in the figure legend, table legend, main text, or Methods section.

n/a	Confirmed	
[ ]	[x]	The exact sample size (n) for each experimental group/condition, given as a discrete number and unit of measurement
[ ]	[x]	A statement on whether measurements were taken from distinct samples or whether the same sample was measured repeatedly
[ ]	[x]	The statistical test(s) used AND whether they are one- or two-sided<br>*Only common tests should be described solely by name; describe more complex techniques in the Methods section.*
[ ]	[x]	A description of all covariates tested
[ ]	[x]	A description of any assumptions or corrections, such as tests of normality and adjustment for multiple comparisons
[ ]	[x]	A full description of the statistical parameters including central tendency (e.g. means) or other basic estimates (e.g. regression coefficient) AND variation (e.g. standard deviation) or associated estimates of uncertainty (e.g. confidence intervals)
[ ]	[x]	For null hypothesis testing, the test statistic (e.g. F, t, r) with confidence intervals, effect sizes, degrees of freedom and P value noted<br>*Give P values as exact values whenever suitable.*
[x]	[ ]	For Bayesian analysis, information on the choice of priors and Markov chain Monte Carlo settings
[x]	[ ]	For hierarchical and complex designs, identification of the appropriate level for tests and full reporting of outcomes
[x]	[ ]	Estimates of effect sizes (e.g. Cohen's d, Pearson's r), indicating how they were calculated

Our web collection on statistics for biologists contains articles on many of the points above.

Software and code

Policy information about availability of computer code

Data collection	RNA sequencing reads were mapped to the M. musculus reference genome GRCm38 using STAR. ChIP-seq reads were aligned uniquely to the mouse genome (mm10) using bowtie (version 1.0.0). Bound regions were detected using MACS2 (version 2.0.10.20131216). GREAT was used for assigning genomic regions to genes. HOMER (version 4.7) was used for obtaining statistics on ChIP enrichment of genome features. Mass Spec - For protein identification, Proteowizard was used to convert raw files to mzXML and searched using X!Tandem (Craig and Beavis, 2004; Kessner et al., 2008) against Human RefSeq Version 45.
Data analysis	Flow cytometry: FlowJo v10 was used for analysis. Expression analysis: GSEA algorithm and analysis using ClusterProfiler package for R, g: Profiler (a web server for functional interpretation of gene lists). ChIP sequencing: Genomation package for R. BioID: The raw data was processed using the CRAPome<br>server. Associations between hits were categorized based onmolecular function using ShinyGO and STRING. Interaction networks were prepared in Cytoscape.

For manuscripts utilizing custom algorithms or software that are central to the research but not yet described in published literature, software must be made available to editors and reviewers. We strongly encourage code deposition in a community repository (e.g. GitHub). See the Nature Portfolio guidelines for submitting code & software for further information.

Data

Policy information about availability of data

All manuscripts must include a data availability statement. This statement should provide the following information, where applicable:

- Accession codes, unique identifiers, or web links for publicly available datasets
- A description of any restrictions on data availability
- For clinical datasets or third party data, please ensure that the statement adheres to our policy

BioID - Repository: available at massIVE (https://massive.ucsd.edu) with access code ID MSV00089158
ChIP sequencing - GEO accession number: GSE200389.
RNA sequencing (ER-Hoxb8 cells): GEO accession number: GSE200391.
RNA sequencing (Fetal liver derived HSPC): GEO accession number: GSE200392
The data is all publicly available. Release date - May, 26, 2023.

Research involving human participants, their data, or biological material

Policy information about studies with human participants or human data. See also policy information about sex, gender (identity/presentation), and sexual orientation and race, ethnicity and racism.

Reporting on sex and gender	There was no consideration of sex or gender in the analysis of human data. PDX mouse data were used in the analysis (figure S14C), but the identity, gender, and ethnicity of the samples were kept confidential.
Reporting on race, ethnicity, or other socially relevant groupings	As above
Population characteristics	NA
Recruitment	There was no recruitment of participants.
Ethics oversight	The approval was obtained from the ethics commission of the Canton of Zurich

Note that full information on the approval of the study protocol must also be provided in the manuscript.

Field-specific reporting

Please select the one below that is the best fit for your research. If you are not sure, read the appropriate sections before making your selection.

☒ Life sciences ☐ Behavioural & social sciences ☐ Ecological, evolutionary & environmental sciences

For a reference copy of the document with all sections, see nature.com/documents/nr-reporting-summary-flat.pdf

Life sciences study design

All studies must disclose on these points even when the disclosure is negative.

Sample size	We calculated the sample size for experiments involving mice using power analysis. We used the freely downloadable software G Power (Faul, Erdfelder, Lang and Buchner, 2007).
Data exclusions	In the immunophenotyping analysis, only GFP-positive ERG-transduced cells were analyzed .
Replication	The results in this manuscript are summary of repeated mice experiments. In the mice experiment presented in figure 3A, six batches of fetal liver derived hematopoietic stem and progenitor cells were used over a two-year period. In the Sknol in vivo experiments, two batches were used for both experiments (HDAC3 enzymatic inhibition and CRISPR-dCAS9 targeting) over a two-month period. There were no unsucssesful randomizations. The replication attempts were all successful for the fetal liver derived and SKNO1 experiments.
Randomization	Each batch of fetal liver derived hematopoietic stem and progenitor cells was split equally for several experimental groups. Control group was included in each batch. Mice were marked and transplanted randomly by technician who was blind to experimental parameters. HDAC3 inhibition in mice transplanted with ERG dependent and dependent cell lines was randomised in a similar manner to the fetal liver experiment.
Blinding	All transplantations were done by a technician who was blind to group allocation.<br>Flow cytometry data collection and analysis were performed by the first co-authors and were not blinded.

Reporting for specific materials, systems and methods

We require information from authors about some types of materials, experimental systems and methods used in many studies. Here, indicate whether each material, system or method listed is relevant to your study. If you are not sure if a list item applies to your research, read the appropriate section before selecting a response.

Materials & experimental systems

n/a	Involved in the study
☐	☒ Antibodies
☐	☒ Eukaryotic cell lines
☒	☐ Palaeontology and archaeology
☐	☒ Animals and other organisms
☒	☐ Clinical data
☒	☐ Dual use research of concern
☒	☐ Plants

Methods

n/a	Involved in the study
☐	☒ ChIP-seq
☐	☒ Flow cytometry
☒	☐ MRI-based neuroimaging

Antibodies

Antibodies used

Western blotting:
ERG (ab133264 -abcam), β-Actin (#3700p -CST), Total H3 (ab1791 -abcam)
Co-IP antibodies:
ERG (sc-271048), NCoR2 (sc-32298), and anti-HDAC3 (sc-11795).
flow cytometry antibodies:
Differentation of human leukemia call lines upon HDAC3 inhibition, Dilution 1:100:
CD13 PE BD 347406
CD34 PerCPCy5.5 BD 347222
CD117 PECy7 BD 333950
CD33 APC BD 345800
HAL-DR PB450 Biolegend 307633
CD45 KO500 BC B36294
CD36 FITC BC IMO766U
CD64 PE BC IM3601U
CD14 APC H7 BD 641394
Propagation of human leukemia cell lines in immune-deficient mice:
hCD45 #17-0459-42, APC, Bioscience (thermofisher).
Fetal liver derived HSCP - immune phenotype:
Alexa Fluor® 700 anti-mouse Ly-6A/E (Sca-1) Antibody, clone D7, cat # 108141, Biolegend
PE/Cyanine7 anti-mouse CD117 (c-Kit) Antibody, clone 2B8, cat# 105813,Biolegend
APC anti-mouse CD150 (SLAM) Antibody, Clone W19132B cat # 162603, Biolegend
Pacific Blue™ anti-mouse CD150 (SLAM) Antibody, clone TC15-12F12.2, cat# 115923, Biolegend
PE anti-mouse CD41 Antibody clone MWReg30 cat# 133905, Biolegend
APC anti-mouse CD61 clone 2C9.G2 (HMβ3-1) Antibody cat# 104315, Biolegend
PE anti-mouse/human CD11b Antibody, clone M1/70, cat # 101207, Biolegend
APC anti-mouse Ly-6G/Ly-6C (Gr-1) Antibody, clone RB6-8C5, cat # 108411, Biolegend
PE anti-mouse CD34 Antibody Clone SA376A4, cat # 152203. Biolegend
APC Annexin V, cat # 640919, Biolegend

Validation

All antibodies were previously published and established. No new antibody/clone was used in this research
Validation of flow cytometry reagents by manufacturers:
Specificity testing of 1-3 target cell types with either single- or multi-color analysis (including positive and negative cell types). Once specificity is confirmed, each new lot must perform with similar intensity to the in-date reference lot. Brightness (MFI) is evaluated from both positive and negative populations. Each lot product is validated by QC testing with a series of titration dilutions.

Eukaryotic cell lines

Policy information about cell lines and Sex and Gender in Research

Cell line source(s)

HEK-293T cells - purchased from ATCC.
Estrogen-regulated ER-Hoxb8 granulocyte-monocyte murine progenitors (GMP) were kindly provided by Prof. David Sykes at the Massachusetts General Hospital.
SKNOI, AML2, AML3, THP1, TFI, ELF153 - were kindly provided by Dr. Michael Milyavsky, Tel Aviv university.
These lines were purchased by Dr. Milyavsky from DSMZ.
THP1 was given from John Dick lab to Dr. Milyavsky.
HNT-34 and MOLM-13 were purchased and maintained by the Zuber lab. The reference was added to the text [46].

	The Bourquin Laboratory generated and maintained TCF3-HLF PDXs.
Authentication	All cell lines were authenticated using STR analysis.
Mycoplasma contamination	All cell lines tested negative for mycoplasma contamination
Commonly misidentified lines (See ICLAC register)	No misidentified lines were used in the study

Animals and other research organisms

Policy information about studies involving animals; ARRIVE guidelines recommended for reporting animal research, and Sex and Gender in Research

Laboratory animals	Immune deficient NSG female mice of 5-8 weeks were used for transplantations of SKNOI cells. C57B/6 female mice of 5-8 weeks were used for transplantations of fetal liver derived HSPC
Wild animals	The study did not involve wild animals.
Reporting on sex	Findings were not related to gender.
Field-collected samples	The study did not involve samples collected from the field.
Ethics oversight	Animal studies were approved by Tel-Aviv University and Chaim Sheba Medical Center at Tel Hashomer Institutional Animal Care and Use Committees. The Helsinki ethics committee at the Chaim Sheba Medical Center authorized all in-vivo experiments (authorization number: 14-051M)

Note that full information on the approval of the study protocol must also be provided in the manuscript.

Plants

Seed stocks	NA
Novel plant genotypes	NA
Authentication	NA

ChIP-seq

Data deposition

- [x] Confirm that both raw and final processed data have been deposited in a public database such as GEO.
- [ ] Confirm that you have deposited or provided access to graph files (e.g. BED files) for the called peaks.

Data access links<br>*May remain private before publication.*	GEO accession number: GSE200389.
Files in database submission	GSM6032522 empty_H3K4me1, BW GSM6032523 empty_H3K4me3 , BW<br>GSM6032524 empty_H3K9Ac, BW<br>GSM6032525 empty_H3K27me3, BW<br>GSM6032526 empty_H3K27Ac , BW<br>GSM6032527 ERG_wt_H3K4me1 , BW<br>GSM6032528 ERG_wt_H3K4me3, BW<br>GSM6032529 ERG_wt_H3K9Ac, BW<br>GSM6032530 ERG_wt_H3K27me3 , BW<br>GSM6032531 ERG_wt_H3K27Ac, BW<br>GSM6032532 ERG_P199L_H3K4me1, BW<br>GSM6032533 ERG_P199L_H3K4me3 , BW<br>GSM6032534 ERG_P199L_H3K9Ac, BW<br>GSM6032535 ERG_P199L_H3K27me3, BW<br>GSM6032536 ERG_P199L_H3K27Ac,BW<br>GSM6032537 lgG_pool
Genome browser session (e.g. UCSC)	https://genome .ucsc.edu/s/Leukem iaGenomics/2019 Eitan_Hox8bmGM P_ C h ıPseq

Methodology

Replicates	ChIP material was prepared from ER-Hoxb8 cells stably expressing ERG variants and an empty vector for control. In each sample, 10A 7 cells were used in duplicates.

Sequencing depth	Median sequencing depth was ~25 million reads per sample, read length was 50 bp, single end. Mapping of reads to the genome resulted in ~ 9% Treads being mapped.
Antibodies	Anti-histones:<br>1. H3K4me1<br>2. H3K4me3<br>3. H3K27Ac<br>4. H3K27me<br>5. H3K9ac - ChIP Grade (ab4441)<br>6. As a control, nonspecific rabbit IgG (15006; Sigma Aldrich) was used.
Peak calling parameters	For the analysis, the following commands and parameters were applied-<br>Data quality<br>1. Read trimming and filtering - cutadapt with the following parameters -<br>-a AGATCGGAAGAGCACACGTCTGMCTCCAGTCAC -a A{60}-a T{60}-0 5 -m 30 -q 10 -e 0.1 --times 2<br>2. Mapping - Bowtie with the following parameters -<br>-m 1 --phred33-quals -5 0 -3 0 --best - 1 -1 --28 nstrata -B 1-5<br>3. Peak calling - MACS2 with default parameters, using input sample as control (with -c parameter)<br>Samtools was used to index the bam files from bowtie and the bw (bigwig) files are from the MACS2 output.<br>Bedtools was used for merging the peaks files and count reads onthem.
Data quality	The default MACS2 peaks filter criteria is a q-value below 0.05, FDR of 5%. The fold enrichment cutoff was 2 fold.
Software	GREAT was used for assigning genomic regions to genes. HOMER (version 4.7) was used for obtaining statistics onChIP enrichment of genome features. Heatmaps and metagene plots of the genes with changed H3K27ac read coverage were plotted using the R package "Genomation" (Akalin et al., 2015). g: Profiler was used for functional enrichment analysis (Raudvere et al., 2019)

Flow Cytometry

Plots

Confirm that:

- [x] The axis labels state the marker and fluorochrome used (e.g. CD4-FITC).
- [x] The axis scales are clearly visible. Include numbers along axes only for bottom left plot of group (a 'group' is an analysis of identical markers).
- [x] All plots are contour plots with outliers or pseudocolor plots.
- [x] A numerical value for number of cells or percentage (with statistics) is provided.

Methodology

Sample preparation	Flow cytometry was used in determination transfection/ transduction efficiency, for cell count, and for immunophenotyping. Hematopoietic tissues (Spleen and Bone marrow (BM)) were harvested from mice at sacrifice time and kept throughout the processing time on ice. BM cells were flashed from the hind leg bones and strained through a 70µm mesh cell strainer. Spleens were mashed on a 70µm mesh cell strainer. Spleens were subjected to red blood cell lysis (Biolegend, San Diego, CA, USA) per manufacturer's protocol. Cells that were not used for immediate analysis/sort were viably frozen in FBS+10% DMSO. Standard staining protocols were used for analysis of cells. In brief, cells were washed in staining media (2%FBS in PBS) and re-suspended in of staining media containing fluorochrome-conjugated antibodies. All cell mixtures were stained with an appropriate color of Fixable Viability Dye (Life Technologies) to exclude dead cells from analysis. Following staining, cells were washed and analyzed.
Instrument	Analysis was done using Gallios flow cytometer (Beckman-Coulter, California, USA). Sorting (For ERG transduced fetal liver derived HSPC cells prior to RNA collection for sequencing) was done using ARIA I/Aria III FACS sorter (BD Biosciences, San Jose, CA USA).
Software	Analysis was performed with FlowJo software (BD).
Cell population abundance	We performed fluorescence-activated cell sorting to isolate the relevant cell populations, and the post-sort fractions were analyzed to confirm their purity.<br>The sorted populations were re-analyzed using flow cytometry to quantify the abundance of our target cells in the isolated fractions. Each fraction was assessed for the presence of marker proteins associated with our cell populations of interest. we consistently achieved purity levels of greater than 95% for our target cells. This was determined by the ratio of marker-positive cells to total cells within the post-sort fraction.
Gating strategy	Compensation beads of each fluorophore were used for cytometer setup and compensations. A "live" cell gate was drawn using FMO to cells pre-stained with a LIVE/DEAD fixable viability dye. For immunophenotyping - Gates were set for ERG transduced populations (GFP positive) followed by gates setting for each cell surface marker using FMO staining of each marker.

- [x] Tick this box to confirm that a figure exemplifying the gating strategy is provided in the Supplementary Information.
